# Stomal Closure: Strategies to Prevent Incisional Hernia

**DOI:** 10.3389/fsurg.2018.00028

**Published:** 2018-04-04

**Authors:** Rhiannon L. Harries, Jared Torkington

**Affiliations:** Department of Colorectal Surgery, University Hospital of Wales, Cardiff, United Kingdom

**Keywords:** ostomy reversal, incisional hernia, stoma site herniation, stoma closure, stoma closure site hernia, Ileostomy, colostomy, hernia

## Abstract

Incisional hernias following ostomy reversal occur frequently. Incisional hernias at the site of a previous stoma closure can cause significant morbidity, impaired quality of life, lead to life-threatening hernia incarceration or strangulation and result in a significant financial burden on health care systems Despite this, the evidence base on the subject is limited. Many recognised risk factors for the development of incisional hernia following ostomy reversal are related to patient factors such as age, malignancy, diabetes, COPD, hypertension and obesity, and are not easily correctable. There is a limited amount of evidence to suggest that prophylactic mesh reinforcement may be of benefit to reduce the post stoma closure incisional hernia rate but a further large scale randomised controlled trial is due to report in the near future. There appears to be weak evidence to suggest that surgeons should favour circular, or “purse-string” closure of the skin following stoma closure in order to reduce the risk of SSI, which in turn may reduce incisional hernia formation. There remains the need for further evidence in relation to suture technique, skin closure techniques, mechanical bowel preparation and oral antibiotic prescription focusing on incisional hernia development as an outcome measure. Within this review, we discuss in detail the evidence base for the risk factors for the development of, and the strategies to prevent ostomy reversal site incisional hernias.

## Introduction

The term “stoma” is derived from the Greek word meaning “mouth” or “opening” and is used to describe the creation of an artificial opening made into a hollow organ, on the surface of the body. A stoma is also sometimes known as an ostomy. The first recorded surgical creation of a stoma was by a French surgeon Pillore in 1776. Within gastrointestinal surgery, stomas are most commonly formed as either an ileostomy or colostomy. These may be permanent or temporary and may be formed from either the end of the bowel or the side of the bowel still in continuity (known as a loop stoma). Temporary stomas, which may be reversed at a later date, are most frequently created in order to divert intestinal contents away from a distal anastomosis or obstructing lesion (known as a defunctioning stoma) or prior to a second operation to restore bowel continuity after surgery for complicated diverticular disease, inflammatory bowel disease or in the case of an obstructing cancer. The most frequent temporary stoma is the defunctioning ileostomy and closure involves the complete freeing of the bowel from all layers of the surrounding abdominal wall, followed by the anastomosis of both the proximal limb and distal limb of bowel to restore bowel continuity. The resultant defect in the abdominal wall is repaired by closure of the musculoaponeurotic layer. Stoma closure can be associated with significant morbidity, including anastomotic leak, obstruction, wound dehiscence, wound infection and the development of an incisional hernia (1–4).

Incisional hernias are defined as “abdominal wall defects, with or without a bulge, around postoperative scars, perceptible or palpable by clinical examination or imaging” according to the European Hernia Society (5). Incisional hernias are common following abdominal surgery, and are likely to develop in the early post-operative period due to separation of the aponeurotic edges. Incisional hernias can cause significant morbidity including pain, altered body image, impaired quality of life (6) and patients can potentially suffer from life-threatening hernia incarceration (6–15%) or strangulation (2%), for which emergency surgery may be necessary (7). Not only do incisional hernias have a significant effect on the patients who suffer with them, but they also pose a significant financial burden on health care systems (8). Repair of incisional hernias (both elective or emergency) are performed frequently, with a total of 12,433 incisional hernia repairs being performed in England alone during 2015–16 (9). The cost per primary incisional hernia repair has been estimated at roughly $16,000 in the United States (10) and 6451€ in France (11).

## Methods

The following sources were searched, Cochrane Library, Ovid, Embase, and Medline using PubMed, with the search terms “ileostomy”, “colostomy”, “ostomy”, “stoma”, “reversal”, “closure”, “hernia”, “incisional”, “surgical site infection” singly or in combination. We supplemented these sources with hand searchs of selected articles and clinical trials registries to find relevant articles.

## Incisional Hernias Following Stoma Closure: How Big Is the Problem?

A meta-analysis published in 2012 investigated the incidence of incisional hernia following closure of stoma, and included 34 studies and a total of 2,729 unique stoma closures (12). Of the stoma closures included, the majority as anticipated were loop ileostomies (47.6%), with the commonest indication for stoma formation being related to colorectal cancer (60.8%). The overall mean incisional hernia rate following stoma closures was 7.4%, with a wide variation across the studies from 0–48% with a median follow-up duration of 36 months. The authors reported a lower risk of hernia following reversal of ileostomy when compared to colostomy on meta-analysis, 4 vs 10% respectively (Odds Ratio (OR) 0.28, 95% CI 0.12–0.65; *p* = 0.003). The reoperation rate for incisional hernia at a previous stoma site was extracted from 12 of the included studies, and was found to be 4%. A further systematic review found a similar incidence for stoma site incisional hernias to be 8.3% (0–33.9%) (13). However, when the authors only included studies with a low risk of bias the incidence was closer to 30%.

Two factors should be noted with regard to the incidence of stoma site hernia. Firstly, that the long-term risk is not known and secondly, that clinical examination alone is shown to have a lower detection rate of incisional hernia post stoma closure when compared to clinical imaging (14, 15). Therefore, studies focusing on only clinical examination may be underestimating the prevalence, as radiological detected herniae may become symptomatic over time and may be missed in studies with a short follow-up period.

## What Are the Risk Factors for the Development of Incisional Hernia Post Stoma Closure?

There is a plethora of evidence describing risk factors for incisional hernia formation, and these can be broadly classified as either patient associated factors or surgical related factors. Patient associated factors include advancing age, male gender, high body mass index (BMI), cachexia, smoking, diabetes mellitus, immunosuppression, glucocorticosteroid use, oral anticoagulants, connective tissue disorders, jaundice, respiratory disorders, and known malignancy (16–28). Many of these patients associated factors may not be correctable prior to surgery. Surgical related factors include emergency surgery, contaminated surgery, abdominal distension, return to theatre, multiple operations, post-operative respiratory failure, suture choice and closure technique (7, 16, 29–32). Most of these risk factors act by either increasing the risk of surgical-site infections (SSI) and wound dehiscence, or by delaying the normal wound healing process (33, 34). Most of the evidence for risk factors comes from work on midline incisions. Studies focusing on incision hernias following stoma closure are few in number, and will be discussed further.

Cingi *et al* (2008) reported a cohort study of 66 patients who had stoma reversal (35). Patients who had incisional hernia at the laparotomy wound were found to have an increased risk of having an incisional hernia at the stoma closure site (OR 4.4). The study found no difference in the development of incisional hernias at stoma closure site when comparing age, gender, BMI or time to stoma closure. Guzmán-Valdivia (2008) found an increased risk of incisional hernia at stoma site in patients with concomitant disease such as diabetes, COPD and hypertension, *p* = 0.005, however numbers in each arm were small (11/22 *cf.* 12/48 without concomitant disease) (36).

Schreinemacher *et al* (2011) performed a retrospective cohort study of 150 consecutive patients who underwent stoma reversals over a 4 year period (37). The authors found a hernia prevalence of 32.4% within the cohort. The only significant risk factor found to be predictive of incisional hernia at stoma closure site was obesity (OR 5.53; 95% CI 1.72–17.80). Gender, age, ASA status, COPD, underlying disease indication for stoma, peritonitis at time of stoma formation, length to stoma reversal or suture material for musculoaponeurotic layer were not found to be predictive risk factors. A further case-control study found that malignancy and diabetes were independently predictive of incisional hernia at stoma closure site (OR 21.93, 95% CI 1.58–303.95 and OR 20.98, 95% CI 3.23–136.31, respectively) (38).

Liang *et al* (2013) compared rates of SSI in consecutive patients undergoing stoma reversals over a 6 year period (39). In 138 patients, they found that history of fascial dehiscence (OR 16.9, 95% CI 1.94–387), colostomy (OR 5.07, 95% CI 2.12–13.0), thicker subcutaneous fat (OR 2.02; 95% 1.33–3.21) and black race (OR 0.35, 95% CI 0.13–0.86) were independent risk factors on multivariate analysis. Shah *et al* (2015) found the following as risk factors for incisional hernia following stoma reversal at a median of 30 months’ follow-up on retrospective review; age, diabetes, end colostomies, loop colostomies, BMI > 30 and urgent operation (40).

Brook *et al* (2016) examined 193 loop ileostomy reversals in order to predict risk factors for hernia development post stoma reversal (41). The results demonstrated that patients experiencing ileostomy site hernia were more likely to have a higher BMI, higher blood pressure at preoperative assessment, colorectal cancer indication for ileostomy formation or postoperative complication occurrence. Logistic regression estimated that for every one unit increase in BMI there was an increase in the OR of 1.2 times the risk of developing a hernia at the stoma closure site. Significance of hypertension persisted on multiple regression independent of ASA status and BMI (OR 18.3), suggesting an intrinsic association of hypertension and stoma closure site hernia development, which the authors suggest could be due to inappropriate activation of inflammatory cytokines causing disordered wound healing (42). The time to ileostomy reversal, initial operative approach (laparoscopic versus open), initial operative urgency (emergency versus elective), consultant surgeon presence, preoperative stoma marking, patient age, gender, smoking status, chemotherapy and suture material choice for musculoaponeurotic layer were not a predictor of hernia development.

Most of the risk factors identified related specifically to incisional hernia post stoma reversal are uncorrectable. It is therefore imperative that surgical techniques are evaluated to ensure we can optimise each patients risk.

## Suture Techniques to Prevent Incisional Hernia

The strength of any sutured wound increases with a higher suture length to wound length (SL/WL) ratio, and is calculated as below:

(Original length of suture –(Length of suture remnants atthe starting knot+Length of suture remnants at thefinishing knot)) /Length of skin incision

Wound dehiscence is unlikely to occur when there is a higher SL/WL ratio (34). Any stomal closure wound should therefore be closed with a SL/WL ratio of greater than 4 in order to prevent incisional hernia formation.

Continuous and interrupted are two suture methods commonly used for abdominal wall closure. Continuous suture method can be advantageous as it uses less suture material and is quicker to perform, but the strength of the entire closure is reliant on the one knot being secure. A meta-analysis published in 2010 included 14 randomised controlled trials with 7,711 patients examined continuous versus interrupted suture method in elective midline laparotomy closure (16). A reduced incisional hernia rate was seen in those with a continuous suture closure technique at a minimum of 12 month follow-up (OR 0.59; 95% CI 0.43–0.82). The authors noted however, that many of the studies that were included were at high risk of bias due to the use of rapidly absorbable suture material in the interrupted suture intervention arm.

Suture bite size has been evaluated in the setting of midline closure in the STITCH Trial (43). Five-hundred and forty-five patients undergoing midline incisional surgery were randomised to either small bite suture placement (0.5 cm from the wound edge every 0.5 cm along the wound, taking aponeurosis within the bite) or large bite suture placement (1 cm from wound edge every 1 cm along the wound). There was lower incisional hernia rate at 1 year follow-up in the small bite group compared the large bite group (13% vs 21%, OR 0.52; 95% CI 0.31–0.87). The small bite technique was also found to have a higher SL/WL (5.0 vs 4.3; *p* < 0.0001). However, the study was quasi randomised (alternated per calendar week) and only included 1 year follow-up. It should also be noted that differing suture material was used in both groups and that in the smaller bite group, aponeurosis only was closed in the bites, therefore posing further questions as to whether the bite size was responsible for the difference in the incisional hernia rate or whether suture material or layers included in the bite played a role.

Other suturing methods have been proposed for midline closure. The Hughes Abdominal Repair Trial (HART) randomised controlled trial, is comparing a far-near-near-far interrupted suture used alongside a continuous suture compared to continuous suture method alone in patients undergoing midline incisions (44, 45). The trial is due to report on its primary outcome of clinically detected incisional hernia at 12 month follow-up in 2019.

To date there has been little evidence which has focused on suture method in stoma site closure. Efforts should be made to investigate the various suture techniques described above within the setting of stoma closures.

## Can Prophylactic Mesh Placement Reduce the Risk?

There are a wide variety of synthetic meshes that are commercially available for use in hernia repairs, these include non-absorbable (either light weight/large pore size or heavy weight/small pore size), absorbable or biological. Meshes can be placed in several locations, either onlay, retromuscular or preperitoneal. There is also a variety of mesh fixation techniques including, suture fixation (both interrupted or continuous technique and suture material choice), tacking staples, fibrin glue, no fixation or self-fixation meshes. There is a minimal evidence base that adequately examines the use of mesh (or the technique for its placement) for prevention of incisional hernia formation post ostomy reversal, which is discussed below.

A retrospective cohort case control study investigated whether the placement of an onlay prophylactic polypropylene mesh at time of stoma closure reduced the rate of incisional hernia after ileostomy closure (38). The study included 83 cases of ileostomy closure over a 5 year period (47 patients with mesh reinforcement and 36 without) with a median follow-up of 18.2 months (IQR 11.7–30.8 months). The rate of incisional hernia was 6.4% in the mesh group compared with 36.1% in the control group, as detected by combined clinical examination and CT imaging (OR 8.29, 95% CI 2.14–32.08; *p* = 0.001).

There have been a number of concerns raised with the use of prophylactic mesh placement and infection due to wound contamination with enterocutaneous flora. It has been proposed that a biological, collagen-based mesh may be a safer adjunct for use in stoma site closures, as the tissue matrix should become incorporated into the host tissue (46). A blinded, case-matched study assessed the retromuscular placement of bioprosthetic collagen porcine mesh at loop ileostomy reversal (47). Thirty patients received mesh reinforcement, and were compared to 64 control patients who received standard stoma closure without mesh placement. At twelve-month follow-up, the incidence of incisional hernia on both clinical examination and CT imaging was reduced in mesh group, but was not statistically significant (3 vs 16% (*p* = 0.17) and 3 vs 19% (*p* = 0.43) respectively). The ROCSS (Reinforcement of Closure of Stoma Site) trial is a randomised controlled trial within the UK assessing the use of intra-abdominal biological mesh reinforcement in comparison to standard closure of stoma sites (48). Recruitment is complete, with 790 patients randomised, and the trial should be reporting its primary outcome of clinical herniation at 2 year follow-up in 2018.

A feasibility study from The Netherlands has also considered whether mesh placement at the time of temporary stoma formation can prevent incisional hernias following stoma closure (49). Ten patients who underwent low anterior resection received an intraperitoneal composite parastomal mesh reinforcement at time of defunctioning stoma formation. At median follow-up of 26 months (14–29) after stoma reversal, no incisional hernias were detected either clinically or on ultrasound imaging. However, it should be noted that adhesions to the mesh were present in all patients in the study and covered a median of 25% of the mesh surface. Whilst the authors commented that no adhesion related morbidity occurred, one patient required a laparotomy in order to mobilise the bowel at time of reversal due to adhesions at the curled up mesh border. Further studies are certainly required to investigate whether mesh placement at time of stoma formation may be of benefit to prevent incisional hernia following reversal, with larger sample size to assess morbidity related to mesh location. Extraperitoneal placement should also be considered in this setting as it may reduce any potential for mesh-related adhesion morbidity.

It should be noted that in situations of planned elective formation of a defunctioning loop ileostomy, then there is a necessity for early function and no stenosis and we also believe that at the time of subsequent closure that a small hernia facilitates the mobilisation.

## Prevention of Surgical-Site Infection with Skin Closure Methods

Whilst the presence of SSI has not been proven to be a risk factor specifically for the development of an incisional hernia at the site of stoma closure, it is accepted as such in incisional hernias in general (33, 34). Methods to prevent SSI should therefore be welcomed within stoma reversal surgeries, particularly as there is pre-existing contamination of the wound from the open bowel lumen. Secondary intention skin healing, several primary closure methods (“air-tight” primary closure, “loose” primary closure or “delayed” primary closure), or a hybrid method utilising a purse-string suture (also known as “circular” closure) have been suggested as methods to deal with the skin at the stoma site following reversal (50–52).

Three meta-analysis have compared circular closure with primary skin closure following stoma reversal. McCarten *et al* (2013) included 2 randomised controlled trials and 4 case controlled series with a total of 403 patients (53). Circular closure suture technique resulted in an 80% reduction in the rate of SSI when compared to primary closure, 2.4 versus 29.6%, respectively (OR 0.083, 95% CI 0.03–0.21; *p* < 0.001). Patients who had circular closure also reported a greater satisfaction with cosmetic outcome (standard mean difference = 0.47 on a patient self-reported assessment of cosmetic outcome using a ten-point visual analogue scale, 95% CI 0.15–0.79; *p* = 0.005). Sajid *et al* (2014) included 3 randomised controlled trials comparing circular closure versus primary skin closure in ileostomy reversal wounds (54). SSI was found to be lower in circular closure (101 patients) when compared to primary closure (105 patients) (OR 0.10, 95% CI 0.03–0.33; *p* < 0.0001). Hsieh *et al* (2015) performed a meta-analysis of 4 randomised controlled trials with 319 participants (55). Circular closure had a lower incidence of SSI when compared to primary closure (Risk difference 0.25, 95% CI −0.36 to −0.15; *p* < 0.00001) and higher satisfaction with cosmetic outcomes (standard mean difference 0.7, 95% CI 0.13–1.27, *p* = 0.02). The “STOMA” trial, a RCT published in 2017, also found a similar result with higher rates of SSI at 30 days in the primary closure arm compared to circular closure (8/27 (30%) versus 3/34 (8%); *p* = 0.03) (56).

A further systematic review and meta-analysis included original articles (3 randomised controlled trials and 12 retrospective reviews) comparing multiple skin closure methods, including primary closure, primary closure with drain, secondary closure, delayed primary closure, loose primary closure and circular closure with a total of 2,921 patients (57). In multiple treatments meta-analysis (random effects) and sensitivity analysis primary closure and delayed primary closure were ranked the worse choices for skin closure, with circular closure ranked the best choice for skin closure.

Circular closure has been hypothesised to decrease rate of SSI as it provides a route for drainage of wound contaminants whilst also providing a higher degree of wound apposition when compared to secondary intention healing. It should also be noted the primary outcome in all the included studies in the meta-analyses was SSI and not incisional hernia rate and many of the studies included were found to be of moderate to high risk of bias. Further studies should focus on well-conducted randomised controlled trial with outcomes measure of stoma site herniation.

## Does Mechanical Bowel Preparation Play a Role with Surgical-Site Infections?

A mulitcentre retrospective study of 272 children who underwent colostomy takedown evaluated the role of mechanical bowel preparation in SSI rates (58). Polyethylene glycol bowel preparation was administered to 187 children pre-operatively and 85 children were classified as controls as they did not receive mechanical bowel preparation. All patients received intravenous antibiotics. Fifty two percent of those who had mechanical bowel preparation received oral antibiotics, whereas no patients in the control group had oral antibiotics. The incidence of SSI was found to be significantly higher in the mechanical bowel preparation group when compared to the controls (14.4 versus 5.8%; *p* = 0.04).

Shah *et al* (2016) performed a prospective randomised study on mechanical bowel preparation within children undergoing elective bowel resection, including stoma closure (59). Eighteen patients were randomised to receive mechanical bowel preparation and 14 were randomised to receive none. The rate of SSI was 11.1% in the treatment arm compared with 21.4% in the group arm, however, this was not found to be statistically significant (*p* = 0.63). The results did not allow extraction of data specific to the cohort of those who underwent stoma closure only and it should be recognized that the sample size was very small and therefore the results of this randomised controlled trial should be taken with caution. Both of these studies were within the paediatric population, which most often have different underlying pathology responsible for the need for stoma formation and therefore the results may not be applicable in adults. There is evidence to support that the use of preoperative mechanical bowel preparation combined with oral antibiotics reduces surgical site infection after colorectal resectional surgery, however this has not been evaluated within patients for ostomy reversal (60, 61). Well conducted studies evaluating the use of mechanical bowel preparation, with or without the combination of oral antibiotics for stoma reversal should be undertaken.

## Conclusions

Incisional hernias following ostomy reversal occur frequently. Despite this, the evidence base on the subject is limited. Many recognised risk factors for their development are related to patient factors and are not easily correctable. There is a limited amount of evidence to suggest that prophylactic mesh reinforcement may be of benefit to reduce the post stoma closure incisional hernia rate but a further large scale randomised controlled trial is due to report in the near future. There appears to be weak evidence to suggest that surgeons should favour circular, or “purse-string” closure of the skin following stoma closure in order to reduce the risk of SSI, which in turn may reduce incisional hernia formation. There remains the need for further evidence in relation to suture technique, skin closure techniques, mechanical bowel preparation and oral antibiotic prescription focusing on incisional hernia development as an outcome measure.

## Author Contributions

RH and JT wrote the review

## Conflict of Interest Statement

The authors declare that the research was conducted in the absence of any commercial or financial relationships that could be construed as a potential conflict of interest.
